# Evolving demographics of eligible patient population can impact enrollment of a biomarker clinical study

**DOI:** 10.1371/journal.pone.0323187

**Published:** 2025-05-09

**Authors:** Sara A. Vettleson-Trutza, Vanessa K. Pazdernik, Joseph H. Skalski, Melissa R. Snyder, Yifei K. Yang

**Affiliations:** 1 Department of Laboratory Medicine and Pathology, Mayo Clinic, Rochester, Minnesota, United States of America; 2 Department of Quantitative Health Sciences, Mayo Clinic, Rochester, Minnesota, United States of America; 3 Division of Pulmonary and Critical Care Medicine, Department of Medicine, Mayo Clinic, Rochester, Minnesota, United States of America; United States Environmental Protection Agency, UNITED STATES OF AMERICA

## Abstract

In a prospective clinical study to better understand how biological markers can improve diagnosis of and prognosis for asthmatic and atopic conditions, we contacted over 3500 eligible patients and observed noticeable differences in the range of their likelihood to enroll based on gender (3.8–13.4%), race and ethnicity (4.8–29.8%), and distance to study site (1.1–29.2%). Both the eligible patients and enrolled participants exhibited a more diverse racial and ethnic composition compared to local population demographics. Based on the eligible patients that the study team contacted (“eligible patients”, n = 3648) and those who agreed to enroll (“enrolled participants”, n = 454), we analyzed the gender, age, race and ethnicity composition of the groups, together with their proximity to the study site. Living close to the study site was the largest contributor to a patient's decision to enroll for both adults (odds ratio OR: 2.26, 95% confidence interval CI: 1.64–3.15, p < 0.001) and children (OR: 2.59, 95% CI: 1.67–4.41, p < 0.001). We also observed that patients from White and non-Hispanic racial and ethnic background were more likely to participate in the study among both pediatric (OR: 1.51, CI: 0.92–2.62, p = 0.122) and adult patients (OR: 1.81, CI: 1.18–2.89, p = 0.009). Eligible patients of female gender were also more likely to enroll in both adult (OR: 1.53, CI: 1.16–2.05, p = 0.003) and pediatric groups (OR: 2.14, CI: 1.42–3.22, p < 0.001). Overall, the pediatric patients (18 years old or younger) were much less willing to participate in the clinical biomarker study. Nonetheless, as they age, the enrollment likelihood increased accordingly (5 years OR: 1.71, CI, 1.32–2.21, p < 0.001). The eligible patient population of the study reflected the evolving demographics and different disease prevalence for asthma and other allergic diseases in adult and pediatric groups. These factors in turn influenced the composition of the enrolled participants.

## Introduction

Clinical studies on biomarkers are crucial in establishing the clinical validity and utility of specific biomarkers in disease diagnosis, prognosis and treatment monitoring [1]. Although biomarker clinical studies do not always meet the definition of a clinical trial, choosing an appropriate study population requires an even distribution of socio-demographics including age, gender, and race/ethnicity that is representative of the general or local population [2]. The racial, ethnic, and gender compositions of a study population may affect the distribution of a biomarker and its clinical validity [3,4]. For example, in using serum creatine to estimate glomeruli filtration rate, clinical studies including larger cohorts of African American and female participants have revealed different levels of creatinine in the female cohort and African American cohort [5]. Although race and ethnicity should not be treated as biological variables, they are linked with genetic and social-economic factors that may contribute to different biological phenotypes and disease prevalence [6]. Nevertheless, most biomarker studies lack the appropriate controls for racial and ethnic compositions, limiting our understanding and generalization of clinical performance of biomarkers across different patient cohorts. Despite academic, industrial, and governmental initiatives to increase racial and ethnic inclusion in clinical trials and biomarker studies, the gap in representation remains. There are both systemic and individual barriers to enrolling in clinical trials and studies, including but not limited to having inadequate access to healthcare [7,8], lack of awareness [8], and finite financial resources [9]. In addition, mistrust of medical establishment and perception of profit driven pharmaceutical research [10,11] could also dissuade eligible patients from participating in clinical trials.

Chronic inflammatory disorders such as asthma, have been shown to impact women and economically disadvantaged groups at a higher prevalence [12] and require ongoing medical treatment at both primary care and specialty care settings. The disparity in disease prevalence is further confounded by age of the affected populations: male children are more likely to have asthma or exhibit asthma symptoms compared to female children, whereas the trend is reversed in adult [13]. In addition to demographic factors, geographic disparities also exist between rural and urban settings, especially regarding asthma disease management and health outcomes [14]. As the development of asthma and other allergic diseases is a complex interplay of genetic predisposition, environmental exposure, and immune adaptation [15], a diverse study population representative of the local genetics and exposome is important to establish the clinical utility for disease biomarkers. To improve engagement and participation in clinical studies, community based participatory research (CBPR) has been an effective approach to involve community partners in early in the study design [16]. By building public awareness and knowledge, CBPR can address existing health disparities and improve access to healthcare resources [17].

During prospective recruitment for a clinical study to evaluate the performance of circulating blood biomarkers to differentiate and evaluate adult and pediatric patients with asthma from other allergic diseases/atopic conditions, we observed that the enrolled participants comprised primarily patients identifying as non-Hispanic White race/ethnicity despite our concerted efforts to identify and recruit patients from diverse backgrounds. We investigated different demographic and geographic factors that may have impacted an eligible participant's likelihood to enroll in the clinical biomarker study. We compared demographics of eligible patients and enrolled participants. Based on varying enrollment likelihoods across different age, racial/ethnic, and gender groups, we identified several underlying factors contributing to the observed selection biases in the enrolled study cohorts.

## Materials and methods

### Study population and recruitment method

**Fig 1:**
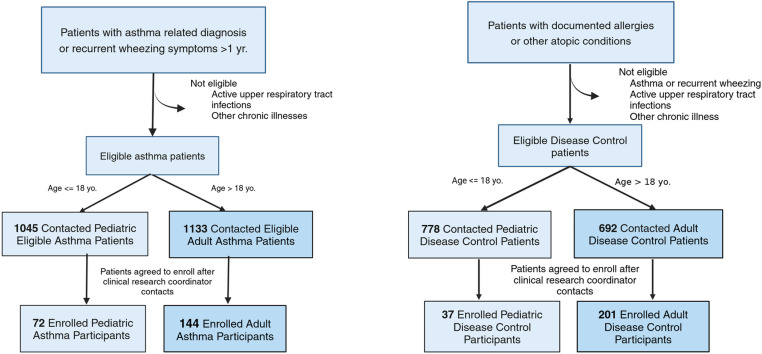
Diagram of the recruitment process, different contacted eligible and enrolled groups. Left: Patients with the pre-defined asthmatic conditions were further refined based on a set of inclusion and exclusion criteria. Based on their age, the eligible asthmatic patients were further divided into adult asthma and pediatric asthma groups. Right: Eligible patients with allergic diseases and other atopic conditions were further divided into adult disease control and pediatric disease control groups. The clinical research coordinators contacted eligible patients from each group and enrolled participants who consented/assented to the study.

Clinical study and patient interactions were carried out according to a study protocol approved by the Mayo Clinic Institutional Review Board (IRB). After receiving IRB approval, recruitment of eligible patients was conducted between October 30, 2021, and March 30, 2023. Data on recruitment outreach and consent events were recorded and extracted from Mayo Clinic Participant Tracking system (PTrax). Ptrax was used to manage the informed consent process, record participant study status, and track enrollments and accruals. Enrollment in this study referred to participants completing a consent form; accruals indicated participants successfully completed their study visit. Eligible patients with diagnosed asthma, or other recorded allergic diseases and atopic conditions were selected based on recorded diagnoses and clinical notes (Fig 1) in their electronic medical records (EMR, Epic Systems), verified by a clinical research coordinator (CRC). Based on the approved protocol (https://www.mayo.edu/research/clinical-trials/cls-20524162), CRCs contacted eligible adults and parents of eligible pediatric patients through their Epic portal or by phone. Study information was also posted on internal employee noticeboards. Patients who expressed verbal interest in participating in the study were contacted again by the CRC to organize an informed consent visit (in-person or virtual). CRCs were aware of participants’ time constraints in scheduling an informed consent during typical working hours, especially in pediatric populations. Accommodations to hold consent visits outside of typical working hours were made whenever feasible. For adult participants a consent visit could be held in person, or virtually through zoom or phone call. For pediatric participants a consent visit required at least one parent or legal guardian to be present and had to be conducted face-to-face which could be fulfilled in person or remotely via a virtual zoom video call. During the consent process, CRCs ensured that the potential participant understood study details, had time to ask questions and upon no further concern completed consent/assent documentation, if interested in participating. A study visit was scheduled upon completion of the consent/assent documentation.

After de-identification, demographic information including gender, race, ethnicity, age, and the last three digits of zip codes of all contacted eligible patients and enrolled participants were collected for study analysis. Demographic data was extracted from PTrax, which collected recorded demographics from EMR. Race groups included American Indian/Alaska Native, Asian, Black or African American, Native Hawaii/Pacific Islander, White, and Other. Ethnicity classifications were non-Hispanic and Hispanic. In our analysis, Non-White race groups and Hispanic ethnicity were combined and classified as non-White groups for statistical comparison purposes. Although both sex and gender information were recorded in EMR, PTrax recorded gender information, and the gender classifications in PTrax included female, male, and unknown/unspecified. As the study paradigm was behavioral in nature [18], we elected to use gender for analysis.

### Study protocol and design

For eligible asthma patients, the inclusion criteria were confirmed diagnosis of asthma or recurrent wheezing symptoms for over one year (Fig 1). The exclusion criteria included active upper respiratory infections (e.g., COVID-19, flu, etc.) and other chronic illnesses. The patient was further classified as pediatric if their age was less than or equal to 18 years old (“Pediatric Asthma patients”, Fig 1). Enrolled participants who had given their informed assent/consent (“Enrolled Pediatric Asthma participants” and “Enrolled Adult Asthma participants”) were asked to 1) provide blood draws for biomarker evaluation; 2) complete an asthma control test questionnaire (ACT) to self-evaluate the severity of their asthma-related symptoms [19]; and 3) perform an optional breath test to measure fractional exhaled nitric oxide (FeNO) to assess airway inflammation [20]. FeNO evaluation was not offered to participants with asthma under the age of 7 due to difficulty in completing the breathing task. For participants with documented allergy or other atopic conditions (Eligible Disease Control patients), inclusion criteria included a recorded allergy or other atopic condition(s) in their EMR. Exclusion criteria included concurrent diagnosis of asthma, recurrent wheezing and/or being actively sick with upper respiratory infections and chronic illnesses. For the disease control group, both adult (“Enrolled Adult Disease Control”) and pediatric participants (“Enrolled Pediatric Disease Control”) were asked to provide blood draws for biomarker evaluation (Fig 1).

In addition to recorded clinical diagnoses and symptoms, additional consideration was given during the screening and contact with pediatric patients under or equal to the age of 18. Based on the approved IRB protocol, it was important for pediatric patients, together with their parents, to be present during the consent process. Their understanding of the study involvement was imperative, and extra care and time was taken to explain the study in an age-appropriate vocabulary (S1 Fig). This was especially important for eligible patients under the age of 7. Youth patients needed to show sufficient understanding of the study or willingness to participate in the study through verbal cue or affirmative language, such as “yes” or “I understand” during the consent conversation before they could be enrolled. Extra care was also taken during the consent conversation to ensure that the parents of these potential participants fully understood their child's involvement. Extra time was spent discussing the blood draw and FeNO test to ensure the parent was comfortable with this study requirement.

The study offered participants a fixed remuneration for their completed study activity to help cover any potential fees for travel to and parking at the study site, as well as to time spent at the study appointment. Due to resource limitations, eligible patients requiring an interpreter for contact and enrollment; residing over 3 hours driving radius of Mayo Clinic Rochester; and patients without sufficient cognitive ability to understand and complete the consent process, were excluded from the group of eligible patients.

### Geographic area

Recruitment was centered within a 3-hour driving radius of the Mayo Clinic in Rochester, Minnesota. The primary focus was on communities surrounding the study site, and encompassed the tri-state area of Minnesota, Wisconsin, and Iowa. The zip codes of residing areas were used to determine distance to study site. Study activities including blood draws and FeNO testing were performed at Mayo Clinic in Rochester, Minnesota. The enrolled participants needed to travel to the primary study site to complete these required evaluations.

### Statistical analysis

All demographic characteristics were summarized as 1) means and standard deviations (SDs) for continuous variables; 2) frequencies and percentages for categorical variables. Chi-square and t-tests were used to discern differences in demographics between cohorts (Table 1). We estimated the local population of the study site (zip codes within 50-mile radius of Rochester, MN) based on the most recent U.S Census Bureau estimates in 2020 [21]. The census data categorized age groups more broadly than the study's distinction between adults and pediatrics (18 years old). To best align with the study's definition, the census summary statistics for the 18–19 age group were included in the adult population for these summaries (S1 Table). We added corresponding 95% confidence intervals (CI) to summaries for gender, racial and ethnic memberships. To evaluate the likelihood to enroll in pediatric and adult groups, we used a multivariable logistic regression model to assess the independent effects of gender, ethnicity/race (“White, non-Hispanic” or “non-White or Hispanic”), and traveling distance (within 50 miles versus beyond) while also adjusting for disease group and age. In the logistic regression model, we adjusted the unit of age to enhance interpretability. For adults, we expressed age in 10-year increments, while for pediatric participants, we used 5-year increments. We grouped American Indian, Asian, Black, Pacific Islander and Hispanic groups together as the “non-White or Hispanic” group to ensure sufficient participants and patients for statistical analysis (Table 1). A full logistic regression model comprising both adults and pediatrics was used to test whether female was more strongly associated with likelihood to enroll in pediatric patients compared to adult while adjusting for disease state, location, and ethnicity/race. Higher-order models were explored through backward elimination starting with all possible 2-way interaction effects for adult and pediatric participants (S2 and S3 Tables). All analyses were conducted using R Statistical Software (v4.2.2; R Core Team 2022) and SAS Studio (v3.82; SAS Institute Inc.). Statistical significance was defined as p < 0.05.

**Table 1:** Demographics and geographic locations of eligible patients and enrolled participants. The patients were grouped based on disease state and age.

	Asthma						Disease control					
	Adult			Pediatric			Adult			Pediatric		
Demographic	Enrolled Participants (n = 144)	Total Eligible (n = 1133)	P	Enrolled Participants (n = 72)	Total Eligible (n = 1045)	P	Enrolled Participants (n = 201)	Total Eligible (n = 692)	P	Enrolled Participants (n = 37)	Total Eligible (n = 778)	P
Gender			0.313^1^			**< 0.001** ^**1**^			**< 0.001** ^**1**^			**0.026** ^**1**^
Female	104 (72.2%)	777 (68.6%)		36 (50.0%)	298 (28.5%)		160 (79.6%)	487 (70.5%)		15 (40.5%)	195 (25.1%)	
Male	40 (27.8%)	356 (31.4%)		36 (50.0%)	747 (71.5%)		41 (20.4%)	204 (29.5%)		22 (59.5%)	583 (74.9%)	
Age			0.700^2^			**< 0.001** ^**2**^			**0.031** ^**2**^			0.149^2^
Mean (SD)	55.0 (16.5)	55.5 (18.5)		11.4 (3.3)	9.2 (3.7)		48.9 (18.2)	51.5 (20.2)		8.6 (4.8)	7.6 (4.3)	
Range	19 - 90	19 - 101		2 - 18	1 - 18		21 - 90	19 - 99		2 - 18	0 - 18	
Ethnicity/Race			0.319^1^			0.613^1^			0.133^1^			0.339^1^
White, Non-Hispanic	132 (93.6%)	980 (87.7%)	**0.023** ^**1**^	58 (80.6%)	788 (77.0%)	0.461^1^	177 (91.2%)	593 (88.8%)	0.197^1^	32 (86.5%)	663 (86.8%)	0.957^1^
Non-White or Hispanic	9 (6.4%)	137 (12.3%)		14 (19.4%)	235 (23.0%)		17 (8.8%)	75 (11.2%)		5 (13.5%)	101 (13.2%)	
American Indian/Alaskan Native	1 (0.7%)	5 (0.4%)		0 (0.0%)	3 (0.3%)		0 (0.0%)	5 (0.7%)		0 (0.0%)	4 (0.5%)	
Asian	0 (0.0%)	18 (1.6%)		2 (2.8%)	39 (3.8%)		9 (4.6%)	22 (3.3%)		0 (0.0%)	31 (4.1%)	
Black or African American	4 (2.8%)	49 (4.4%)		4 (5.6%)	88 (8.6%)		2 (1.0%)	22 (3.3%)		1 (2.7%)	29 (3.8%)	
Native Hawaii/Pacific Islander	0 (0.0%)	1 (0.1%)		0 (0.0%)	0 (0.0%)		0 (0.0%)	0 (0.0%)		0 (0.0%)	0 (0.0%)	
Hispanic or Latino	3 (2.1%)	54 (4.8%)		7 (9.7%)	68 (6.6%)		5 (2.6%)	22 (3.3%)		2 (5.4%)	24 (3.1%)	
Other	1 (0.7%)	10 (0.9%)		1 (1.4%)	37 (3.6%)		1 (0.5%)	4 (0.6%)		2 (5.4%)	13 (1.7%)	
Location			**< 0.001** ^**1**^			**0.023** ^**1**^			0.733^1^			**< 0.001** ^**1**^
Beyond 50 miles	47 (32.6%)	619 (54.6%)		25 (34.7%)	498 (47.7%)		16 (8.0%)	59 (8.5%)		3 (8.1%)	267 (34.3%)	
Within 50 miles	97 (67.4%)	514 (45.4%)		47 (65.3%)	547 (52.3%)		185 (92.0%)	633 (91.5%)		34 (91.9%)	511 (65.7%)	

1. Pearson’s Chi-squared test; 2. t-test.

## Results

### Enrolled participants and their demographics

In the enrolled adult asthma group (Fig 1, n = 144), most participants identified as White non-Hispanic. Only 5.7% of the enrolled adult participants were from American Indian/Alaska Native, Black or African American, or Hispanic groups (Table 1). The average age was 55, with a wide range (19–90). Most adult participants with asthma were female (72.2%). The majority of the adult participants with asthma (67.4%) resided within 50 miles of the clinical evaluation site. There were fewer participants enrolled in the pediatric asthma group (n = 72), with an average age of 11.4 (range 2–18). Compared to adults, there were significantly more pediatric participants (18%, p < 0.05) from Asian, Black, or African American, and Hispanic or Latino groups. The gender distribution was even between female and male among pediatric participants with asthma.

In the Disease Control Group, there were 37 pediatric participants (Enrolled Pediatric Disease Control) and 201 adult participants (Enrolled Adult Disease Control) respectively (Fig 1 and Table 1). Among adults, the average age was 48.9 (range 21–90). Female participants represented 79.6% of the enrolled adults. A large proportion of the adult participants (91.2%) identified as White and non-Hispanic. Only 8.2% of enrolled adult Disease Control participants came from Asian, Black, or African American, and Hispanic or Latino groups. Most enrolled participants resided within 50 miles of the study evaluation site (92.0%). We observed similar trends in the Pediatric Disease Control Group, where most of the participants were White and non-Hispanic (86.5%). Nevertheless, male and female participants were more evenly distributed among the pediatric group, at 59.5% and 40.5% respectively. Pediatric participants ranged from 2–18 yo., with an average age of 8.6.

### Racial and ethnic demographics of the local population

Based on the most recent US census data, the local population living within 50 miles of the study site (Rochester, MN) is primarily White and non-Hispanic, comprising 92.9% of the total population (S1 Table). The second largest racial and ethnic group of the region is Hispanic or Latino (3.2%), followed by Black or African American (1.9%), Asian (1.8%), American Indian/Alaskan Native (0.2%), and Native Hawaii/Pacific Islander (0.05%). Largely agricultural, the surrounding areas are rural and sparsely populated [22], except for the urban center of Rochester city. Similar to national trends, the local population has become more racially and ethnically diverse. In the youth population (<=17 yo.), there is higher share of non-white groups (11.7%) compared to the population of people 18 or older (5.8%). Relative to local demographics, our enrolled participants consisted of larger proportions of participants from non-White racial and ethnic backgrounds (S2 Table). Nevertheless, such comparison did not take into consideration the different prevalences of asthma and atopic conditions in different racial and ethnic groups. Asthma is a heterogenous disease with varying prevalences among different racial and ethnic groups, impacted both by genetics and exposure history. Black individuals and indigenous people are shown to have the highest asthma rates compared to other races and ethnicities, followed by non-Hispanic White individuals [23]. Although Hispanic population as a whole show lower asthma disease prevalence, Puerto Ricans residing in the continental United States have the highest asthma rate up to 19.0% [24].

### Eligible patients for study recruitment

At the conclusion of patient recruitment, similar numbers of eligible pediatric (Fig 1, Eligible pediatric asthma group n = 1045) and adult (Eligible adult asthma group, n = 1133) asthma patients were contacted by the CRC for recruitment. For the disease control cohorts, we contacted more pediatric patients (Eligible pediatric disease control group, n = 778) with documented allergy and atopic conditions, compared to the eligible adult patients (Eligible adult disease control group, n = 692).

Among all contacted eligible adult patients with asthma (n = 1133), patients from non-White racial and ethnic groups represented 12.3% (Table 1). The majority were female (68.6%), and the average age of the group is 55.5 (range: 19–101). Compared to adult participants with asthma, a larger proportion of the eligible patients from the pediatric asthma group (23.0%) were from American Indian, Asian, Black, Pacific Islander and Hispanic groups. Conversely, there were significantly more male patients (71.5%) in the eligible group compared to the female (28.5%). The average age of the eligible pediatric group was 9.2 (range: 1–18). There were more eligible pediatric patients living within 50 miles of the study site (52.3%). Relative to the local population within 50 miles, there were more non-White or Hispanic eligible patients in both the adult and pediatric cohorts (9.7% [95% CI, 7.3 to 12.7] adults; 29.5% [95% CI, 25.6 to 33.5] pediatrics) (S2 Table).

In the Contacted Adult Disease Control group (n = 692), we observed a similar trend of eligible patients reporting predominantly White and non-Hispanic ethnicity (Table 1, 88.8%). They were also more likely to be female (70.5%) and lived close to the study site (91.5%). The pediatric eligible patients have similar racial and ethnic make-up (86.8% White, non-Hispanic). Most of the eligible pediatric patients were male (74.9%) and lived within 50 mile of the study site (65.7%). The average age of the pediatric eligible group is 7.6 (range: 0–18). The eligible patients within 50 miles of the study site comprised more patients from non-White or Hispanic backgrounds compared to the local population demographics.

### Likelihood to enroll in clinical studies

**Fig 2:**
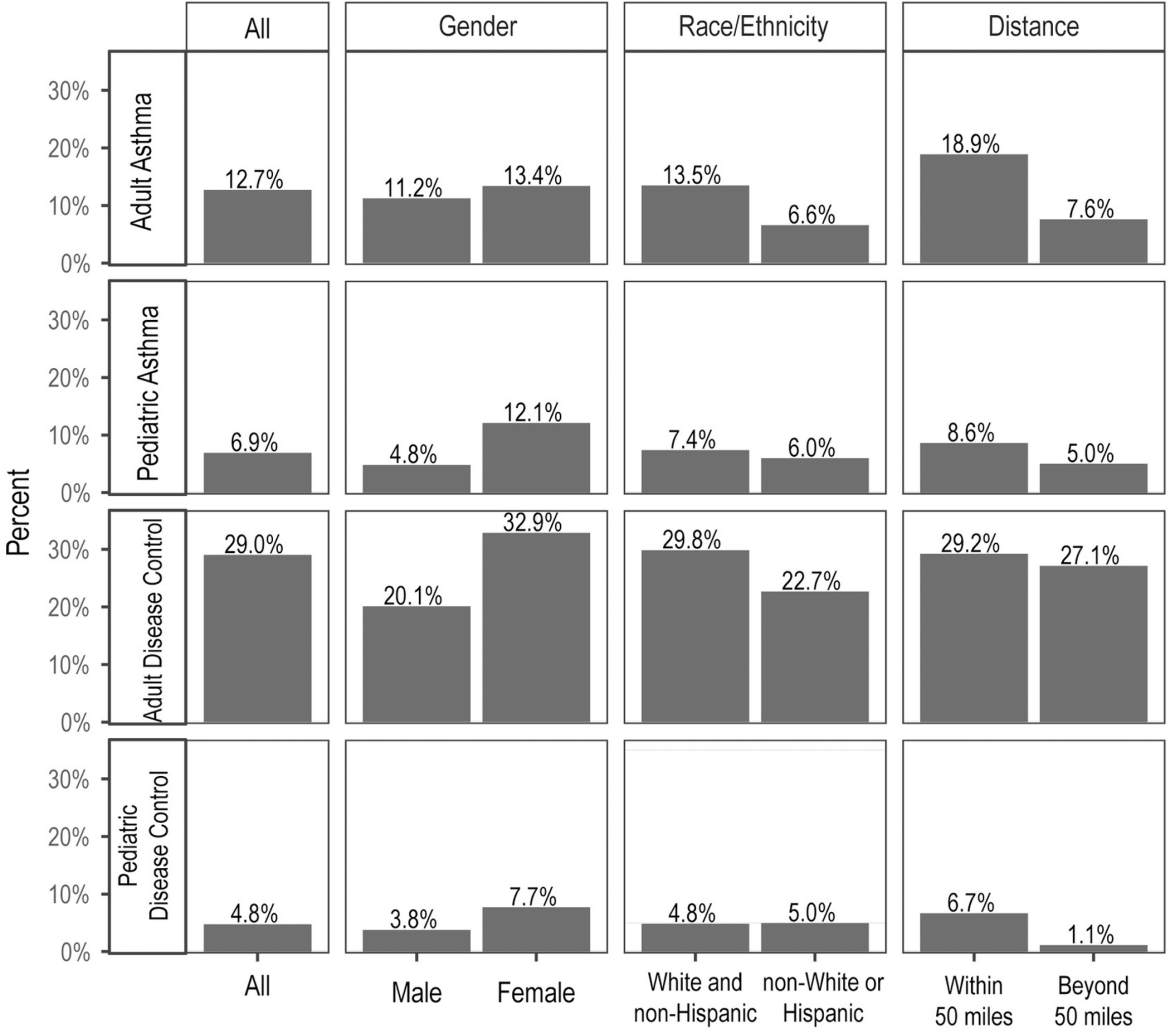
Enrollment likelihood of eligible patients. The participation was calculated based on gender, race and ethnicity groups, and distance to the study site.

**Fig 3:**
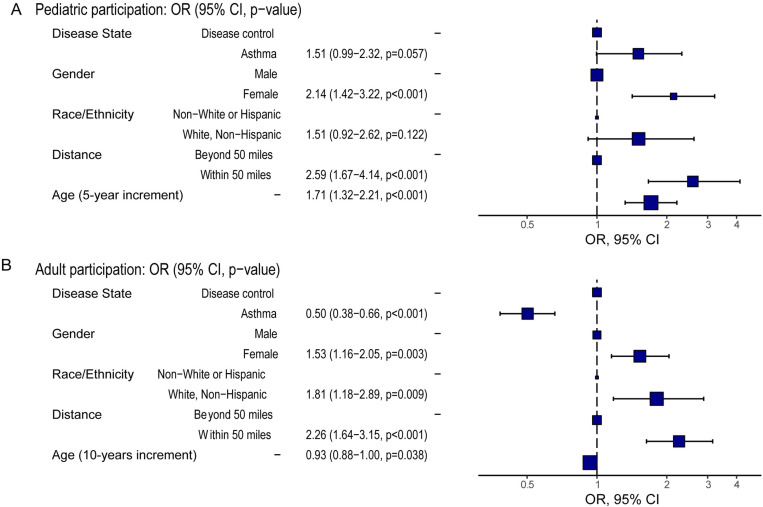
Odds ratios for study enrollment. A) Pediatric participation based on a multivariable logistic regression. The age increment unit was set at 5 years. B) Participation in the adult group, variables include disease state, gender, race/ethnicity, distance to study site, and age increment at 10 years.

We were able to identify factors impacting a person's likelihood to enroll by comparing demographics between the enrolled and contacted groups. In general, adult patients were more likely to enroll in the study compared to pediatric patients (Fig 2), observed in both the asthma and disease control cohorts. Across all age and disease cohorts, more female patients chose to participate in the clinical study (Fig 2). Adjusting for other demographic factors and disease conditions, female pediatric participants were more likely to participate (OR: 2.14, CI: 1.42 to 3.22; p < 0.001) compared to pediatric male patients (Fig 3). In adult participants, the adjusted odds ratio was 1.53 (CI, 1.16 to 2.05; p = 0.003). This gender difference was significantly larger in pediatric compared to adult participants (p = 0.02). Among adult participants, the adjusted odds ratio for obtaining participation from White, non-Hispanic patients was 1.81 (CI: 1.18 to 2.89; p = 0.001) compared to their counterparts in non-White groups (Fig 3). The effect was in the same direction for pediatric patients but not statistically significant (p = 0.122). Proximity to study location impacted patients’ decision to participate in the clinical study too. Patients living close to the primary study site (within 50 miles) were significantly more likely to participate. After adjustment for other demographic factors and disease conditions, odds ratios were similar across pediatric (OR = 2.59; CI, 1.67 to 4.14; p < 0.001) and adult participants (OR = 2.26; CI, 1.64 to 3.15; p < 0.001) (Fig 3). In the study, we didn't reimburse study participants differently depending on their travel distance.

Higher-order models were explored to examine interactions between demographic factors, traveling distance, and disease states in both adult and pediatric patients. For adults, the final model identified interactions between distance and both gender (p = 0.004) and disease states (p = 0.02) (S3 Table). Female participation was higher among those living within 50 miles of the site (female vs male: OR = 1.94; CI, 1.25, 3.02; p < 0.001), while such effect was not observed among eligible patients living beyond 50 miles (female vs male: OR = 0.76; CI, 0.37, 1.57; p = 0.76). Longer traveling distance was a deterrent to participate among adults with asthma, having a lower likelihood to enroll for those living beyond 50 miles (asthma, beyond vs within 50 miles: OR = 0.43; CI, 0.26, 0.73; p < 0.001), but not for eligible patients in the disease control group (beyond vs within 50 miles: OR = 1.03; CI, 0.45, 2.35; p > 0.99). Among pediatric participants, an interaction between age and disease group was identified (S4 Table, p = 0.008). Age was positively associated with enrollment in the asthma group (5-year increment: OR = 2.25; CI, 1.60 to 3.17; p < 0.001) but not in the disease control group (5-year increment: OR = 1.13; CI, 0.77 to 1.67; p = 0.53). There was also a higher enrollment of White, non-Hispanic female participants. Overall, gender and distance to the study site were the most significant factors influencing enrollment decisions.

## Discussion

### Changing demographics of eligible patients impact enrollment decisions

We observed a more diverse pediatric patient population in our study, reflecting the shifting national demographic trend [21]. In pediatric groups with asthma and other atopic conditions, the proportion of eligible patients from Hispanic or Latino, Black or African American, and Asian backgrounds were substantially higher compared to the adult groups (Table 1). Although the manifestations vary, atopic conditions, including asthma share similar over-activated immunoglobulin-E (IgE) responses. The development of certain atopic conditions, such as eczema and food allergies, can occur during early childhood [25]. In comparison, the development of asthmatic symptoms and disease diagnosis tend to occur later in childhood [26]. The average age of the eligible pediatric asthma group (9.2 yo.) is slightly higher compared to the eligible pediatric disease control group (7.6 yo., Table 1). Based on our analysis, age is a crucial determinant of a patient's likelihood to enroll, especially in the pediatric population. During study recruitment, we did contact more pediatric eligible patients compared to adult patients in our recruitment process. A pediatric participant's decision to enroll was dependent on both their willingness and parents/legal guardian's interest. Conversely, the decision not to participate could also be attributed to either. Although we were not able to pin down specific reasons, during our conversation, parents of very young children (less than 5 yo.) were often less willing to subject their children to an unnecessary needle stick. In our study, the likelihood for eligible pediatric asthmatic patients to participate grew two-fold with every 5-year increase in age. The study protocol required a face-to-face (in person or virtual video) participation appointment for pediatric study participants in addition to the in-person study visit for sample accrual. For the study visits including a blood draw and lung function test, the child needed to be accompanied by their parent/legal guardian to the study site. As a result, a child's likelihood to participate in this clinical study also reflected the economic resources of their caregivers. For both adult and pediatric participants, living close to the study site (within 50 miles) was associated the closest with their willingness to enroll (Fig 3).

### Gender, race and ethnicity are associated with varying likelihood of enrollment

Female patients were more likely to participate in the study compared to their male counterparts, similarly to other published studies [27,28]. In the pediatric population, male children are known to more likely to develop atopy, including asthma compared to female children [15,29], accordingly we observed a higher proportion of male eligible patients in our contacted cohorts for both asthma (71.5%) and atopy disease control groups (74.9%). Female pediatric patients were also twice (OR: 2.14) as likely to participate in the asthma biomarker study, resulting in a more even gender distribution in the enrolled groups (Table 1). For adults, the trend for asthma and other atopic diseases was reversed, and there were more female eligible patients in both the asthma and disease control groups. Female adult patients were more likely to participate in the study compared to male (OR: 1.53). As a result, the enrolled adult participants were skewed towards females (Table 1).

Race and ethnicity further modified the demographic composition of the enrolled participants. In both adult and pediatric patients, we observed a higher enrollment rate among non-Hispanic White patients. In pediatric patients, the differences were not statistically significant due to limited numbers of patients from non-White or Hispanic backgrounds. Our study population was focused on patients residing within 3-hour driving distance of the study site, encompassing a largely rural area. Furthermore, diagnosis and treatment for atopic conditions and asthma routinely occur in primary care settings where patients utilize the most accessible healthcare resources [30]. Although the eligible adult patients we contacted were comprised of higher percentages of patients from non-White backgrounds compared to our local population, these patients were less likely to participate. There have been confounding results in published studies investigating racial and ethnic disparities in clinical trials [3,21,22]. Our study further highlighted the potential barriers facing under-represented patient populations when deciding to enroll in a clinical study.

### Strategies to improve participation and their feasibilities

To improve engagement and participation in the biomarker study, we explored several outreach strategies to engage under-represented patient groups, particularly for the pediatric patients. The study team presented and discussed our study with the pediatric outreach board, a community-based collaboration between local youth volunteers and the institution, to learn more about alternative outreach strategies [31]. Although direct interactions with potential pediatric patients through social media and school information forums, such as peach jar [32], may have further raised the profile of the study, our institutional IRB generally did support commercial strategies to promote clinical trials. As travel distance to the study site was identified as a main determinant of the patient's enrollment decision, we provided virtual appointments for participation/assent and flexible hours whenever feasible for the CRCs. We explored de-centralized options to complete study activities, such as completing an electronic questionnaire about disease severity [33]. However, the blood biomarkers and lung function tests requiring an in-person visit limited our options.

Although Mayo Clinic has long-standing relationships with local communities based on diagnosis and treatment of serious and complex diseases [34], the institution primarily serves as an international referral center for specialty care. By partnering with local hospitals to provide community health resources such as education materials and free periodic health screenings, we may further build trust among local communities, especially patients from under-represented ethnic and racial groups [35]. In addition, by engaging patients in the decision-making process of clinical research, we can employ CBPR methods to empower community members and in-turn improve their access to healthcare resources [36–39].

### Study limitations

The recruitment and enrollment data were collected during a prospective clinical study evaluating the diagnostic utility of blood-based biomarkers in assessing asthma and other allergic diseases. The unbalanced numbers in contacted patients and enrolled participants were the results of different enrollment likelihoods among patient groups. Although our analysis revealed certain variables associated with different odds ratios for study participation, we could not verify the exact reasons when an eligible patient (adult or pediatric) decided to participate or not. In addition to the factors considered in our study, other factors such as social-economic status and cultural influences can also impact a patient's enrollment decision. We did not have access to that information, hence cannot analyze or comment further. Because there were very few patients who identified as American Indian/Alaskan native, Asian, Black or African American, and Hispanic or Latino in our dataset, we had to combined them into one group “Non-White or Hispanic” for statistical analysis, thus creating an oversimplified dichotomy.

## Conclusions

Our study highlighted practical challenges to recruit and enroll a more diverse patient population for biomarker studies. There is growing awareness and interest to participate in clinical studies among younger generations, who are also increasingly diverse in their racial and ethnic make-up. Several demographic factors impacted the study populations, and the study team may need to over-sample specific demographic groups to counter their varying likelihood of enrollment.

## Supporting Information

S1 Fig Assent form for pediatric participants. Languages were adapted based on age to ensure understanding.
(PDF)

S1 Table Demographics of local population (within 50 miles). The estimation was based on the most recent US census data (2020). The surrounding area is largely rural.
(XLSX)

S2 Table Demographics of contacted eligible participants who resided within 50 miles. The dataset included eligible patients residing within 50 miles of the study site, further divided based on disease and age groups.
(XLSX)

S3 Table Higher-order interactions between different factors for enrollment among adults. Logistic regression from backward elimination model selection predicting participation among eligible adults.
(XLSX)

S4 Table Higher-order interactions between different factors for enrollment among children. Logistic regression from backward elimination model selection predicting participation among eligible pediatrics.
(XLSX)

S5 Table Anonymized dataset for enrollment participants. (CSV)

## Abbreviations

PTrax: Participant Tracking system
EMR: electronic medical records
CRC: Clinical research coordinator
ACT: asthma control test questionnaire
FeNO: fractional exhaled nitric oxide
OR: odds ratio
CI: confidence interval
SD: standard deviation
IRB: Institutional Review Board
CBPR: Community based participatory research
