# Supplementary material for: Evolving demographics of eligible patient population can impact enrollment of a biomarker clinical study
Source: PLoS One. 2025 May 9;20(5):e0323187. doi: 10.1371/journal.pone.0323187 (PMC12063902; doi:10.1371/journal.pone.0323187)
Supplement: S1 Fig — Languages were adapted based on age to ensure understanding. (PDF) [file pone.0323187.s001.pdf]

Name and Clinic Number
**Protocol #: 21-008894**<br>**Subject ID:**

Assent Form to Take Part in a Research Study

TITLE: Clinical Utility of Eosinophil-Derived Neurotoxin (EDN) in Asthma Diagnosis and Evaluation

IRB #: 21-008894

PRINCIPAL INVESTIGATOR:

You are being asked to be in a research study. This study might help us understand asthma

You are going to have some blood taken out of your arm. The person taking your blood will poke a needle in your arm and collect the blood in a tube. The person will only collect about two spoonfuls of blood. The needle poke may hurt, and you will have to sit still for a few minutes. But once you are done, you can go home. We will send you $25.00 in the mail for participating.

You may also be asked to take a test that measures the gases in your breath. This study is voluntary. If you choose to do this test, you will be asked to blow air into a small machine, similar to blowing up a balloon. You do not have to perform this test if you don't want to.

No one will be mad at you if you say no.

I would be willing to have my blood drawn

☐ Yes ☐ No Please initial here: ________Date: ________

I would be willing to participate in a Fractional Nitric Oxide test.

☐ Yes ☐ No Please initial here: ________Date: ________

Name and Clinic Number
**Protocol #: 21-008894**<br>**Subject ID:**

Assent of Child

Child Name Date (mm/dd/yyyy) Time (hh:mm am/pm)

Child Signature

If the child does not sign the form, but you believe the child has actively assented, please document on this form. State the specific behaviors (head shake yes, child said okay after you described the procedure, etc.).

Person Obtaining Consent (Researcher) Date (mm/dd/yyyy) Time (hh:mm am/pm)

Signature
